# Efficient Biotransformation of Icariin to Baohuoside I Using Two Novel GH1 β-Glucosidases

**DOI:** 10.3390/molecules29225280

**Published:** 2024-11-08

**Authors:** Xiaoling Zhang, Yitong Wang, Tiantian Zhang, Ziqiao Yuan, Yongjun Wei

**Affiliations:** 1 School of Pharmaceutical Sciences, Zhengzhou University, Zhengzhou 450001, China; 2 State Key Laboratory of Cotton Bio-Breeding and Integrated Utilization, Zhengzhou University, Zhengzhou 450001, China; 3 Food Laboratory of Zhongyuan, Zhengzhou University, Zhengzhou 450001, China

**Keywords:** biotransformation, icariin, baohuoside I, β-glucosidase, enzyme characterization

## Abstract

*Epimedium Folium* (EF) is a traditional Chinese herbal medicine, and its primary bioactive ingredients, such as icariin, are flavonoid glycosides. A rare EF flavonoid, baohuoside I, exhibits superior bioactivities and enhanced bioavailability compared to its metabolic precursor icariin. The biotransformation of icariin to baohuoside I can be effectively and specifically achieved by β-glucosidases. In this study, 33 candidate full-length β-glucosidase genes were screened from a previously built carbohydrate active enzyme (CAZyme) gene dataset derived from cow fecal microbiota. Thirteen of them exhibited β-glucosidase activity, with DCF-bgl-26 and DCF-bgl-27 showing relatively high expression levels and β-glucosidase activity. The maximum β-glucosidase activity of DCF-bgl-26 and DCF-bgl-27 was achieved at 45 °C and pH 6.0, with DCF-bgl-26 demonstrating better thermostability and pH tolerance compared to DCF-bgl-27. The activities of DCF-bgl-26 and DCF-bgl-27 were 123.2 U/mg protein and 157.9 U/mg protein, respectively, both of which are higher than those of many bacterial β-glucosidases. Structure analysis suggested that both β-glucosidases possess canonical (β/α)_8_-TIM barrel fold structure of GH1 family β-glucosidases. Thin-layer chromatography results showed that both enzymes could efficiently convert icariin to baohuoside I in 30 min, indicating they have potential application in the production of high value rare baohuoside I.

## 1. Introduction

*Epimedium Folium* (EF), known as Yin-Yang-Huo in Chinese, is a traditional Chinese herbal medicine with over 2000 years of use in China and other Asian countries. EF is often prescribed alone or in combination with other herbs to treat various diseases, including kidney-Yang deficiency syndrome [1], osteoporosim [2], rheumatoid arthritis [3], and asthma [4]. It is also frequently used in healthy products, with over 501 healthy foods containing EF currently available on the market [5]. The primary bioactive substances of EF are flavonoid glycosides, including epimedin A, epimedin B, epimedin C, icariin, icariside I, baohuoside I (icariside II), and icaritin [6]. These compounds are formed by varying degrees of glycosylation of the C-3 and C-7 positions of aglycone icaritin [7]. Among these, icariin is one of the most abundant flavonoids in most EF samples (about 1%) [6,8] and is recognized as a quality control standard for EF herbs [9].

Baohuoside I, the direct and main metabolite of icariin [10,11], exhibits multiple bioactivities more potent than icariin [6,12]. It displayed superior efficacy in osteoclastogenesis inhibition and bone resorption suppression in vivo compared to icariin [13]. Additionally, baohuoside I (IC_50_ = 9.2 μM) presented stronger anti-cancer activity against cervical cancer than icariin (IC_50_ > 120 μM) [14]. Furthermore, baohuoside I exhibited higher bioavailability than icariin, due to its lower polarity, which facilitated better absorption by intestinal epithelial cells [15]. However, the content of baohuoside I in EF is very low, about 0.09% in the 30 analyzed EF samples [16]. Due to the attractive pharmaceutical activities and high bioavailability of baohuoside I, efficient production of the rare flavonol glycoside baohuoside I is of significant interest.

Currently, baohuoside I is mainly produced via acid hydrolysis, column chromatography, and microbial transformation. However, these methods have some limitations, including low efficiency, undesirable byproducts, time-consuming, and environmental pollution [17,18]. Alternatively, enzymatic transformation offers unique advantages, including high efficiency, high selectivity, and mild reaction conditions [19], for the direct production of baohuoside I from icariin [12,17,18]. Given that icariin contains an additional glucose group at the C-7 position compared with baohuoside I, the conversion of icariin to baohuoside I can be effectively achieved by using β-glucosidases with high efficiency and specificity to remove the glucose moiety.

β-Glucosidases (EC 3.2.1.21) are a class of glycoside hydrolases (GH), which can hydrolyze the β-glycosidic bond in the non-reducing terminal residue of β-D-glucoside, resulting in the release of glucose [20]. Depending on the sequences of amino acid and structural similarities, β-glucosidases are mainly distributed in the GH1 family and GH3 family [21,22]. The GH1 family includes most of the β-glucosidases that have been identified and characterized so far, and GH1 β-glucosidases are known to be more tolerant to glucose compared to GH3 β-glucosidases [21,23]. Comparative structural analysis suggested a clear correlation between the shape and electrostatic properties of the active site entrance of GH1 β-glucosidases and their glucose tolerance [21,23]. The GH1 family presents a typical (α/β)_8_ TIM-barrel structure and a pocket-like catalytic channel [20,21].

GH1 β-glucosidases have been applied in various fields, including food and beverage production, the textile industry, and trace active compounds transformation [21,22]. However, there are only a few β-glucosidases that have been used to transform icariin to baohuoside I. A thermostable GH1 β-glucosidase IagBgl1 of *Ignisphaera aggregans* exhibited maximum activity at 95 °C and pH 6.5 and could transform icariin into baohuoside I with a molar conversion of 99.48% [12]. Additionally, a GH3 family β-glucosidase Tpebgl3 from *Thermomotoga petrophila DSM* 13,995 was immobilized on Na-Y zeolite, which could hydrolyze icariin to produce baohuoside I, and the molar conversion rate reached to 97.6% at 75 °C and pH 5.0 [24]. Furthermore, a β-glucosidase from *Trichoderma viride* can generate baohuoside I from icariin with optimal efficiency at 41 °C and pH 4.0 [25]. The metagenomic approach has been applied for the discovery of diverse glycoside hydrolases from environments like biogas digester, termite hindgut, and cow rumen, including xylanase [26], β-glucuronidase [27], and pectinolytic enzyme [28]. Considering the excellent glucose tolerance of GH1 β-glucosidase, screening β-glucosidases using metagenome provides a feasible and efficient strategy to obtain novel GH1 β-glucosidases with the desired properties for the transformation of icariin to baohuoside I.

In this study, β-glucosidase genes from previously established metagenomic data of the dairy cows’ fecal microbiota were screened [29]. A total of 33 full-length β-glucosidase genes were identified and expressed in *Escherichia coli*, and their catalytic activity was investigated. Then, the structures of certain bioactive β-glucosidases were analyzed, and their ability of transforming icariin to baohuoside I was evaluated.

## 2. Results and Discussion

### 2.1. Screening of 13 Active β-Glucosidases from Metagenomic Data

A comprehensive dataset comprising over 280,000 carbohydrate-active enzyme (CAZyme) genes had been built from the metagenome of dairy cows’ fecal microbiota [29]. A total of 82 full-length GH1 family genes (Genbank accession numbers from PQ155168 to PQ155249) were identified from the CAZyme gene dataset. After comparing these genes with known β-glucosidase genes in GenBank, 33 genes were identified as potential β-glucosidase genes. The full length of these 33 candidate β-glucosidase genes ranged from 963 to 2328 bp, with protein sequence identities of 68.15–99.78% to known genes in NCBI (Table 1).

**Figure 1:**
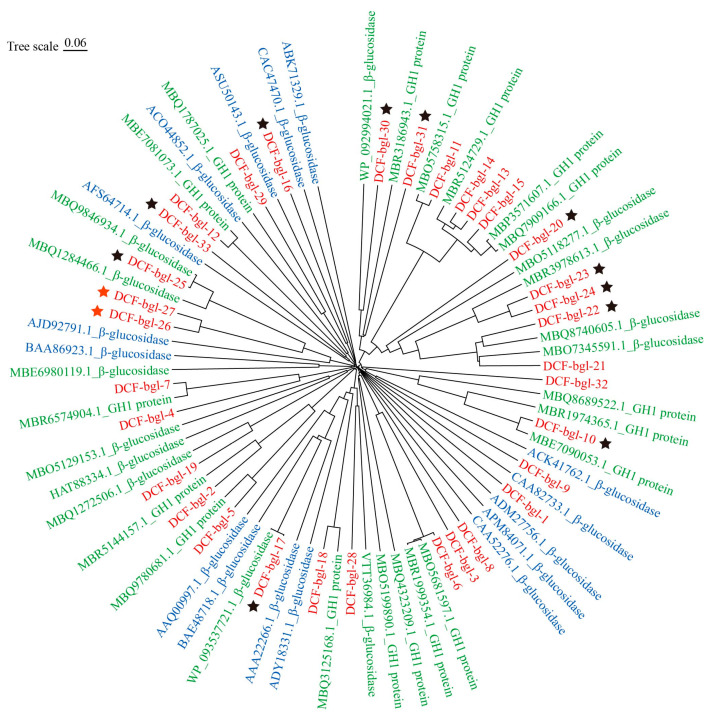
The phylogenetic tree of the 33 predicted β-glucosidases and other β-glucosidase protein sequences. The 33 predicted β-glucosidases were colored with red, the 31 known sequences with high sequence identity from NCBI were colored in green, and the 16 known active β-glucosidases from the CAZy database were colored with blue. The 13 expressed proteins that displayed β-glucosidase activity were marked with stars, and the two that showed high β-glucosidase activity, DCF-bgl-26 and DCF-bgl-27, were marked with red stars.

Some of the 33 selected genes showed high sequence similarity with known but uncharacterized β-glucosidase genes from the ruminant gastrointestinal microbiota, suggesting these genes are likely essential in lignocellulose degradation and conserved in ruminant animals. Phylogenetic analysis showed that the 33 candidate GH1 β-glucosidase genes formed several distinct clusters, differing from the 16 known β-glucosidases in the CAZy database (Figure 1). In particular, the cluster composed of DCF-bgl-11, 13, 14, 15, 20, 21, 22, 23, 24, 30, 31, and other sequences in GenBank, was obviously different from the 16 known characterized β-glucosidases, indicating that novel β-glucosidases were available in the selected 33 β-glucosidase genes.

**Figure 2:**
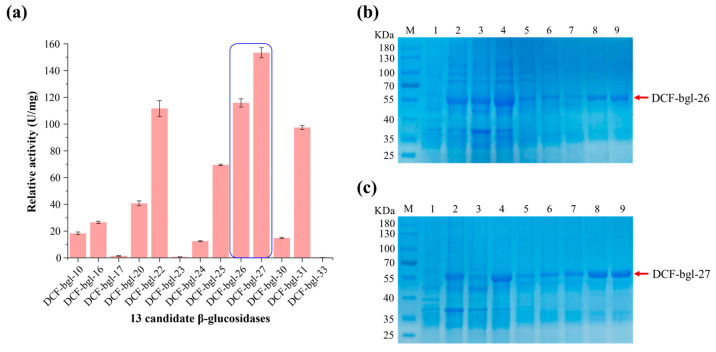
The catalytic activity of the 13 β-glucosidases and SDS-PAGE of DCF-bgl-26 and DCF-bgl-27. (**a**) The relative activity of the 13 β-glucosidases. (**b**) M: protein markers; Lane 1: the protein supernatant of *E. coli* BL21 (DE3) expressed empty plasmid as a negative control; Lane 2: the total supernatant protein mixture of *E. coli* BL21 (DE3) expressed DCF-bgl-26; Lane 3: the precipitated protein mixture of *E.coli* BL21 (DE3) expressed DCF-bgl-26; Lane 4: Ni column flow-through solution after protein absorption; Lane 5: protein sample washed with 20 mM imidazole buffer; Lane 6: protein sample washed with 50 mM imidazole buffer; Lane 7: protein sample washed with 100 mM imidazole buffer; Lane 8: protein sample washed with 300 mM imidazole buffer; Lane 9: protein sample washed with 500 mM imidazole buffer. (**c**) M: protein markers; Lane 1: the protein supernatant of *E. coli* BL21 (DE3) expressed empty plasmid as a negative control; Lane 2: the total supernatant protein mixture of *E. coli* BL21 (DE3) expressed DCF-bgl-27; Lane 3: the precipitated protein mixture of *E.coli* BL21 (DE3) expressed DCF-bgl-27; Lane 4: Ni column flow-through solution after protein absorption; Lane 5: protein sample washed with 20 mM imidazole buffer; Lane 6: protein sample washed with 50 mM imidazole buffer; Lane 7: protein sample washed with 100 mM imidazole buffer; Lane 8: protein sample washed with 300 mM imidazole buffer; Lane 9: protein sample washed with 500 mM imidazole buffer.

The crude activity of the 33 candidate β-glucosidase was tested, and 13 of them (39.40%) displayed β-glucosidase activity (Figure S1). Further β-glucosidase activity detection with *p*NPG as substrate revealed that 2 of the 13 candidate β-glucosidases, DCF-bgl-26 and DCF-bgl-27, exhibited the two highest activities (Figure 2a). Thus, DCF-bgl-26 and DCF-bgl-27 were selected for enzymatic characterization.

### 2.2. Purification and Enzymatic Characterization of DCF-bgl-26 and DCF-bgl-27

The recombinant β-glucosidases DCF-bgl-26 and DCF-bgl-27 with 6-histidine tag at the C-terminus were successfully overexpressed in *E. coli* BL21 (DE3). The theoretical molecular weights of DCF-bgl-26 and DCF-bgl-27 recombinant protein were 52.97 kDa and 52.68 kDa, respectively. The SDS-PAGE analysis showed that both enzymes were highly purified, with their bands appearing around 55 kDa (Figure 2b,c).

**Figure 3:**
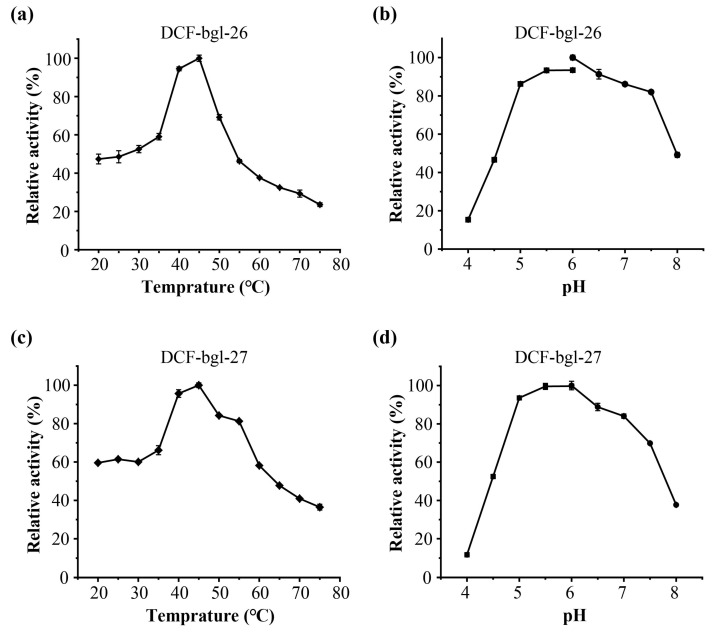
The enzymatic characteristics of DCF-bgl-26 and DCF-bgl-27. (**a**) The optimal temperature of DCF-bgl-26; (**b**) the optimal pH of DCF-bgl-26; (**c**) the optimal temperature of DCF-bgl-27; (**d**) the optimal pH of DCF-bgl-27. These activities were displayed as relative values, and the error bars represented the standard deviation of triplicates.

**Figure 4:**
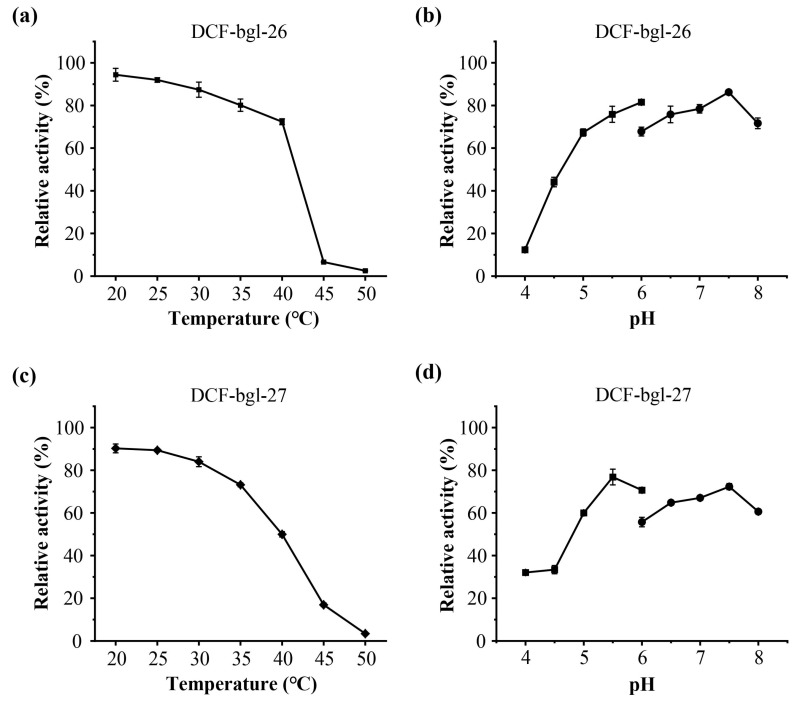
The thermal stability and pH tolerance of DCF-bgl-26 and DCF-bgl-27. (**a**) The thermal stability of DCF-bgl-26; (**b**) the pH tolerance of DCF-bgl-26; (**c**) the thermal stability of DCF-bgl-27; (**d**) the pH tolerance of DCF-bgl-27. The values represented the mean values of triplicates, and the error bars indicated the standard deviation.

The optimal temperature and pH of DCF-bgl-26 and DCF-bgl-27 were 45 °C and pH 6.0 (Figure 3). Their optimal conditions are consistent with the typical feature of GH1 family β-glucosidases, which generally exhibit optimal temperatures at ~50 °C and pH ≥ 6.0 [21]. For temperature stability, DCF-bgl-26 could maintain over 70% activity after incubation at 20 °C to 40 °C for 1 h (Figure 4a), while DCF-bgl-27 could maintain over 50% of its activity after incubation at 20 °C to 40 °C for 1 h (Figure 4c). For pH tolerance, DCF-bgl-26 could keep over 70% of its activity from pH 5.0 to pH 8.0 after 1 h incubation (Figure 4b), while DCF-bgl-27 could keep over 50% of its activity from pH 5.0 to pH 8.0 after 1 h incubation (Figure 4d). They were similar to certain β-glucosidases from bacteria, which were observed to have high activity and stability at a neutral pH range [30].

The activity of DCF-bgl-26 and DCF-bgl-27 for *p*NPG hydrolysis at 45 °C and pH 6.0 were 123.2 U/mg protein and 157.9 U/mg protein, respectively. Their hydrolytic activity on *p*NPG were higher than that of certain bacterial-derived β-glucosidases, such as BglD5 (39.48 U/mg) from *Jeotgalibacillus malaysiensis* [31], BGLA (59 U/mg) from *Alteromonas* sp. L82 [30], and AsBG1 (50 U/mg) from *Alicyclobacillus* sp. A4 [32]. In summary, both DCF-bgl-26 and DCF-bgl-27 were acidic β-glucosidases with mild optimum temperature and high hydrolysis efficiency. Although DCF-bgl-27 exhibited slightly higher activity than DCF-bgl-26, the latter showed better thermostability and pH tolerance.

### 2.3. Bioinformatics Analysis of DCF-bgl-26 and DCF-bgl-27

**Figure 5:**
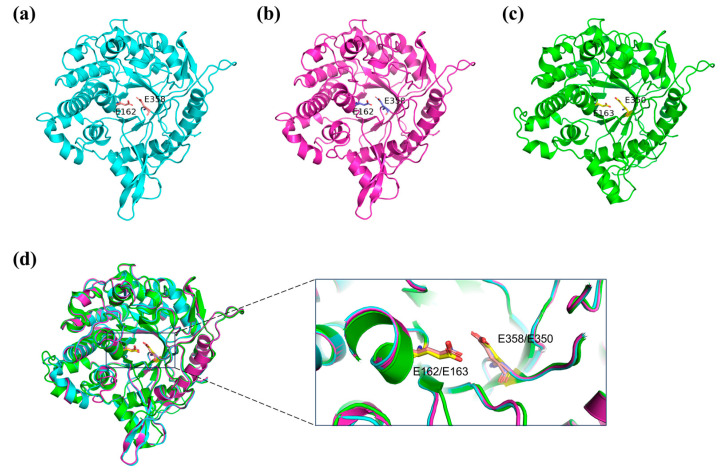
The predicted structures and structure comparisons of DCF-bgl-26 and DCF-bgl-27. (**a**) The predicted cartoon structure of DCF-bgl-26. It exhibited the canonical (β/α)_8_-TIM barrel fold structure and had two catalytic residues, E162 and E358, and the C atoms were colored with orange. (**b**) The predicted cartoon structure of DCF-bgl-27. It had the (β/α)_8_-TIM barrel fold structure and two catalytic residues, E162 and E358, and the C atoms were colored with blue. (**c**) The cartoon structure of Br2 β-glucosidase (PDB: 8J3M), a known GH1 family β-glucosidase. It had the (β/α)_8_-TIM barrel fold structure and two catalytic residues, E163 and E350, and C atoms were colored with yellow. (**d**) The superimposition of DCF-bgl-26 and DCF-bgl-27 with Br2, the three structures and the two catalytic residues were overlapped very well.

Both DCF-bgl-26 and DCF-bgl-27 consist of 453 amino acids and share a protein sequence identity of 87.2%. They exhibited the highest sequence identity with the GH1 β-glucosidase Br2 (PDB ID: 8J3M) from bovine rumen metagenome (Figure 5c) [33]. DCF-bgl-26 showed 49.55% sequence identity to Br2, and DCF-bgl-27 showed 51.58% sequence identity to Br2. The theoretical pI of DCF-bgl-26 and DCF-bgl-27 were 5.23 and 4.99, respectively. There was no signal peptide in the two β-glucosidases predicted by Signal 6.0.

The structures of DCF-bgl-26 and DCF-bgl-27 were predicted with AlphaFold2 (Figure 5a,b). The structural alignment with Br2 suggested that the three enzymes had a similar structure (Figure 5d), exhibiting the canonical (β/α)_8_-TIM barrel fold structure of GH1 family β-glucosidases (Figure 5a,b). Comparison of the sequences of DCF-bgl-26 and DCF-bgl-27 with another 4 known GH1 family β-glucosidases indicated that these two new β-glucosidases had a series of conserved amino acid residues in the substrate binding site (Figure S2). These included two special strictly conserved motifs of NEP (residues 161–163) and TENG (residues 357–360) [23,33]. Among the motif, E162 (acid/base catalyst) and E358 (nucleophile) overlapped well with the catalytic residues E163 and E350 of Br2 (Figure 5d). These results suggested that DCF-bgl-26 and DCF-bgl-27 were GH1 family β-glucosidases.

### 2.4. Enzymatic Transformation of Icariin into Baohuoside I by DCF-bgl-26 and DCF-bgl-27

**Figure 6:**
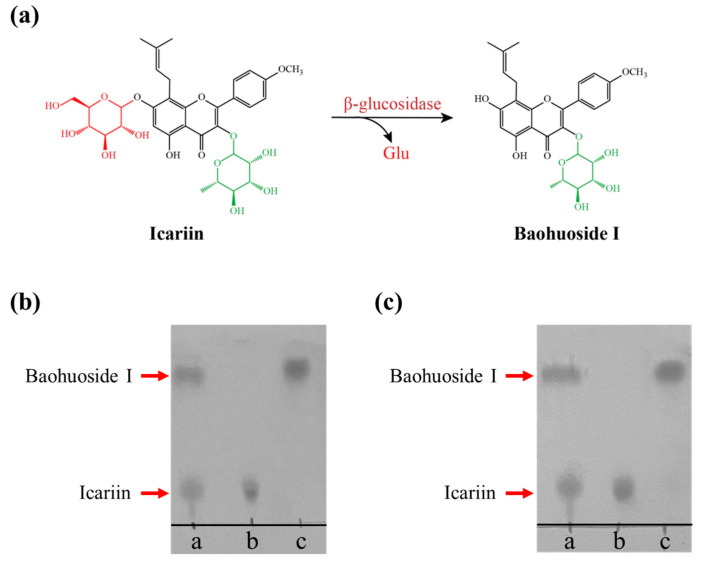
The hydrolysates of icariin catalyzed by DCF-bgl-26 and DCF-bgl-27. (**a**) The catalytic mechanism of β-glucosidase for the conversion of icariin to baohuoside I. (**b**) Line a: the icariin and baohuoside I standard marker; Line b: the hydrolysates of icariin by inactivated DCF-bgl-26 (negative control); Line c: the hydrolysis products of icariin by DCF-bgl-26. (**c**) Line a: icariin and baohuoside I (standard marker); Line b: the hydrolysates of icariin by inactivated DCF-bgl-27 (negative control); Line c: the hydrolysis products of icariin by DCF-bgl-27.

Baohuoside I and icariin share the same flavonoid skeleton, except icariin has one extra glucose group at the C-7 position [7]. Icariin is one of the major flavonoids in EF herbs; however, baohuoside I is a rare component of EF [8,16], though it has attractive pharmaceutical activities and higher bioavailability [6,15]. Therefore, icariin was employed as a potential substrate for producing baohuoside I using β-glucosidase (Figure 6a). Both DCF-bgl-26 and DCF-bgl-27 were able to efficiently convert icariin to baohuoside I in 30 min under optimal temperature and pH conditions, with icariin being almost completely converted to baohuoside I (Figure 6b,c). This indicated that DCF-bgl-26 and DCF-bgl-27 had excellent icariin biotransformation ability. It is noted that the hydrolysis activity of DCF-bgl-26 and DCF-bgl-27 to the standard substrate *p*NPG were higher than IagBgl1 (92.47 U/mg) and Tpebgl3 (95.7 U/mg) [12,24], which have been used to transform icariin to baohuoside I with a high conversion rate. The optimal reaction conditions for DCF-bgl-26 and DCF-bgl-27 (45 °C and pH 6.0) differ from those of the three reported β-glucosidases used for icariin biotransformation [12,24,25]. This offers excellent new β-glucosidase options for the conversion of icariin to baohuoside I. However, further evaluation is needed to accurately assess the biotransformation efficiency of their application in flavonoid glycosides biotransformation. Both enzymes were recovered using a metagenomic approach, which demonstrated its potential application for identifying other industrially valuable or useful enzymes.

## 3. Materials and Methods

### 3.1. Strains, Plasmids, and Reagents

*E. coli* strains TOP10 and BL21(DE3) were purchased from Tolo Biotech Co., Ltd. (Hefei, China). The pET-28a (+) vector was bought from GeneCreate Co., Ltd. (Wuhan, China). The 4-Nitrophenyl-β-D-glucopyranoside (*p*NPG) was purchased from Aladdin Biochemical Technology Co., Ltd. (Shanghai, China). Icariin and baohuoside I were bought from Meilun Biotechnology Co., Ltd. (Dalian, China). Aescin was bought from Yuanye Biotechnology Co., Ltd. (Shanghai, China). Ferric citrate was purchased from Maclean Biochemical Technology Co., Ltd. (Shanghai, China). FastPure Gel DNA Extraction Mini Kit, FastPure Plasmid Mini Kit, and ClonExpress II One Step Cloning Kit were obtained from Vazyme Biotechnology Co. (Nanjing, China).

### 3.2. Screening of Novel β-Glucosidase Genes from Metagenomic Data of Cow Fecal Microbiota

The carbohydrate active enzyme (CAZyme) gene dataset has been built from the metagenomic sequencing data of dairy cows’ fecal microbiota, which was fed with multiple traditional Chinese herbs (Henan Muyi Animal Pharmaceutical Co., Ltd, Kaifeng, China) [29]. The GH1 family predicted to be full-length were screened. Homology analysis of these genes was carried out with the BlastX program on the NCBI website (https://blast.ncbi.nlm.nih.gov/Blast.cgi, accessed on 10 April 2023). Then, the genes with more than 40% similarity to known β-glucosidase genes were selected.

The phylogenetic relationships of the candidate genes were assessed in comparison with 31 similar known genes in GenBank and 16 known β-glucosidases in the CAZy database. Protein sequence alignment and phylogenetic tree construction for these 80 genes were performed by MEGA 11 software with default parameters. The visualization and customization of the obtained phylogenetic tree were carried out using the Chiplot website (https://www.chiplot.online/, accessed on 24 July 2024).

### 3.3. Gene Expression and Catalytic Activity Determination of Candidate β-Glucosidase Genes

A total of 33 primer pairs (Table S1) were used to amplify the 33 predicted β-glucosidase genes. The obtained gene fragments were inserted into the pET-28a (+) vector. These 33 recombinant plasmids were then individually transformed into *E. coli* TOP10 strain, and gene sequence verification was carried out by DNA sequencing. The correct recombinant plasmids were subsequently transformed into *E. coli* BL21 (DE3) strain for protein expression. LB-esculin-agar was used to detect the crude catalytic activity of each candidate β-glucosidase [34]. When *E. coli* BL21(DE3) containing one recombinant plasmid was coated on LB-esculin-agar and incubated at 37 °C for 16 h, if the plate turned black, this suggested that the corresponding expressed protein exhibited glucosidase activity.

### 3.4. Expression and Purification of Active β-Glucosidases

The *E. coli* BL21 (DE3) strains harboring the recombinant active β-glucosidases were inoculated into 100 mL LB medium containing 50 μg/mL kanamycin in a 500 mL shake flask at a 1/100 volume ratio. The cultures were incubated at 37 °C, 200 rpm until the OD_600_ reached 0.6–0.8, then 0.2 M IPTG was added. Following cultivation at 20 °C and 180 rpm for 16 h, the cells were harvested by centrifugation at 8000× *g* rpm for 10 min. The harvested cells were washed 3 times with PBS buffer (pH 7.4) and then resuspended in 50 mL lysis buffer (pH 7.4) containing 1 mM phenylmethylsulfonyl fluoride (PMSF, protease inhibitor).

Cell disruption was performed using an ultrasonic crusher (Xiaomei ultrasonic instrument company, Kunsan, China) on ice at 150 W for 10 min, with 3 s crushing and 5 s interval in lysis buffer (Na_2_HPO_4_-NaH_2_PO_4_ 20 mM, NaCl 300 mM, imidazole 5 mM, at pH 7.4). The cell lysate was then centrifuged at 4 °C and 12,000× *g* for 10 min. The supernatant was collected and purified using Ni-NTA affinity chromatography (Smart-Lifesciences Biotechnology Co., Ltd., Changzhou, China). After the supernatant was added to Ni-NTA column for protein adsorption, wash buffer 1, 2, and 3 (Na_2_HPO_4_-NaH_2_PO_4_ 20 mM, NaCl 300 mM, at pH 7.4, with imidazole 20, 50, or 100 mM, respectively) were used to wash away unabsorbed proteins. Then, elution buffer 1 and 2 (Na_2_HPO_4_-NaH_2_PO_4_ 20 mM, NaCl 300 mM, at pH 7.4, with imidazole 300 or 500 mM, respectively) were used to elute target protein in the sequence. The high-purity protein samples were concentrated with 10 kDa ultrafiltration tubes (Merck, Millipore, MA, USA). The protein purity was assessed by sodium dodecyl sulfate-polyacrylamide gel electrophoresis (SDS-PAGE), and protein concentration was determined using the Bradford method (Sangon Biotech Co., Ltd., Shanghai, China).

The relative activity of the purified β-glucosidases was evaluated by measuring their ability to hydrolyze the standard substrate *p*NPG [35]. The reaction mixture consisted of 20 μL *p*NPG (5.0 mM), 170 μL phosphate buffer (pH 7.4), and 10 μL appropriately diluted β-glucosidase sample, and was incubated at 45 °C for 5 min. The reaction was then terminated by adding 50 μL Na_2_CO_3_ (0.5 M). The OD_405_ value was measured using Spectra MR spectrophotometer (Dynex Technologies, Chantilly, VA, USA) to determine the concentration of produced *p*-nitrophenol (*p*NP). The β-glucosidase activity was calculated using the prepared standard curve. One unit (U) of β-glucosidase activity was defined as the amount of enzyme that released 1 μM of *p*NP per min under the assay conditions.

### 3.5. Effects of Temperature and pH on DCF-bgl-26 and DCF-bgl-27 Activity

To determine the optimal temperature of DCF-bgl-26 and DCF-bgl-27, a 200 µL reaction mixture consisting of 170 μL phosphate buffer (pH 7.4), 20 μL 5 mM *p*NPG, and 10 μL appropriately diluted enzyme solution was prepared. The reaction system was incubated at 20 °C, 25 °C, 30 °C, 35 °C, 40 °C, 45 °C, 50 °C, 55 °C, 60 °C, 65 °C, and 70 °C for 5 min, respectively, and then the reaction was ended by adding 50 μL 0.5 M Na_2_CO_3_ immediately. The OD_405_ value of sample was measured, and the temperature with the highest OD_405_ value was considered as the optimal temperature for the respective enzyme.

To determine the optimal pH of DCF-bgl-26 and DCF-bgl-27, the reaction temperature was fixed at 45 °C, which was identified as the optimal temperature for the two enzymes. Na_2_HPO_4_-citric acid (pH 4.0–6.0, 0.2 M) and Na_2_HPO_4_-NaH_2_PO_4_ (pH 6.0–8.0, 0.1 M) were used for the pH test. The reaction system, reaction time, and β-glucosidase activity detection method (*p*NPG method) were the same as the optimal temperature determination experiment.

The thermostability of DCF-bgl-26 and DCF-bgl-27 was measured separately by incubating enzymes at 20 °C, 25 °C, 30 °C, 35 °C, 40 °C, 45 °C, 50 °C, 55 °C, 60 °C, 65 °C, and 70 °C for 1 h under pH 6.0 (the measured optimal pH for the two β-glucosidases) using 0.1 M Na_2_HPO_4_-NaH_2_PO_4_ buffer. Then, the residual activity of two enzymes was measured at pH 6.0 and 45 °C. The highest β-glucosidase activity without temperature pre-treatment was defined as 100%.

The pH stability was examined by pre-incubation of each enzyme at room temperature for 1 h in a pH range from 4.0 to 8.0, and then the residue activity of DCF-bgl-26 and DCF-bgl-27 with pH pre-treatment was measured at 45 °C and their corresponding pH was determined. The 100% activity was referred to samples without pH pre-treatment. All experiments were repeated three times.

### 3.6. Enzymatic Transformation of Icariin into Baohuoside I

The hydrolytic abilities of DCF-bgl-26 and DCF-bgl-27 on icariin were detected by thin-layer chromatography (TLC) assay. The 100 μL reaction system included 40 μL icariin (0.4 mg/mL), 20 μL purified enzyme (appropriately diluted concentration), and 40 μL phosphate buffer. The reaction was carried out at the optimal temperature and pH for 30 min. Then, 20 μL ethyl acetate was added to extract the products and terminate the reaction, followed by centrifugation at 12,000× *g* rpm for 5 min.

The supernatant was spotted on silica gel plate GF254 (Qingdao Ocean Chemical Co., Ltd., Qingdao, China) in the spread solvent of ethyl acetate:chloroform:formic acid:water (10:1:1:1, by volume). A mixture of icariin and baohuoside I was used as the marker, while the hydrolysates of inactivated enzyme reaction with icariin were used as the control. After the samples spread completely, the developed TLC plate was dried at room temperature and sprayed with 10% ethanol sulfate for coloration. The TLC plate was further heated in an oven at 100 °C for 10 min until brown spots appeared.

### 3.7. Bioinformatics Analyses of DCF-bgl-26 and DCF-bgl-27

The SignalP-6.0 server was used to predict the signal peptide of DCF-bgl-26 and DCF-bgl-27. AlphaFold2 was employed to predict the 3D structures of DCF-bgl-26 and DCF-bgl-27 with default parameters, and PyMOL2.5 was used to visualize and compare the predicted DCF-bgl-26 and DCF-bgl-27 structures with similar known β-glucosidases structures.

## 4. Conclusions

In this study, we screened 82 full-length potential GH1 β-glucosidase genes from a previously established metagenomic dataset of dairy cows’ fecal microbiota. A total of 33 β-glucosidase genes were further identified, cloned, and expressed, of which 13 exhibited β-glucosidase activity, and DCF-bgl-26 and DCF-bgl-27 exhibited the top two activities. Both enzymes were acidic β-glucosidases, displayed identical optimal temperature and pH, and showed high efficiency to transform icariin to baohuoside I. Structure predication indicated that they had the canonical (β/α)_8_-TIM barrel fold structure of GH1 family β-glucosidase. Our study highlighted the effectiveness of the metagenomic strategy in recovering novel glycoside hydrolases and provided two novel efficient β-glucosidases for the conversion of icariin to produce rare baohuoside I. Based on this, β-glucosidases can be used to construct engineered bacteria, especially engineered probiotics, which could ferment EF efficiently, offering a promising direction for the full utilization of EF in the near future.

## Figures and Tables

**Table 1:** The 33 predicted β-glucosidase genes and their most closely related sequences in GenBank.

33 Candidate β-Glucosidase Genes	Blast Alignment Results	The Alignment Sequence ID	Identity
DCF-bgl-1	Beta-glucosidase [*Treponema* sp.]	MBO5118277.1	95.31%
DCF-bgl-2	Glycoside hydrolase family 1 protein [*Clostridia* bacterium]	MBR5144157.1	85.47%
DCF-bgl-3	Glycoside hydrolase family 1 protein [*Clostridia* bacterium]	MBO5199890.1	69.72%
DCF-bgl-5	Beta-glucosidase [*Oscillospiraceae* bacterium]	MBO5129153.1	74.28%
DCF-bgl-4	Glycoside hydrolase family 1 protein [*Clostridia* bacterium]	MBQ9780681.1	84.24%
DCF-bgl-6	Glycoside hydrolase family 1 protein [*Lentisphaeria* bacterium]	MBR1999354.1	99.54%
DCF-bgl-7	Glycoside hydrolase family 1 protein [*Clostridia* bacterium]	MBR6574904.1	93.49%
DCF-bgl-8	Glycoside hydrolase family 1 protein [*Lentisphaeria* bacterium]	MBO5681597.1	75.52%
DCF-bgl-9	Family 1 glycosyl hydrolase [*Clostridia* bacterium]	MBQ8689522.1	80.05%
DCF-bgl-10	Glycosyl hydrolase family protein [*Clostridiales* bacterium]	MBE7090053.1	94.04%
DCF-bgl-11	Family 1 glycosyl hydrolase [*Clostridia* bacterium]	MBO5758315.1	96.82%
DCF-bgl-12	Glycosyl hydrolase family protein [*Clostridiales* bacterium]	MBE7081073.1	94.56%
DCF-bgl-13	Family 1 glycosyl hydrolase [*Clostridia* bacterium]	MBR5124729.1	93.20%
DCF-bgl-14	Family 1 glycosyl hydrolase [*Clostridia* bacterium]	MBP3571607.1	93.03%
DCF-bgl-15	Family 1 glycosyl hydrolase [*Clostridia* bacterium]	MBQ7909166.1	91.46%
DCF-bgl-16	Family 1 glycosyl hydrolase [*Clostridia* bacterium]	MBR1974365.1	87.39%
DCF-bgl-17	GH1 family beta-glucosidase [*Psychrobacillus psychrotoler*]	WP_093537721.1	99.55%
DCF-bgl-18	Family 1 glycosyl hydrolase [*Clostridia* bacterium]	MBQ3125168.1	88.14%
DCF-bgl-19	Glycoside hydrolase family 1 protein [*Clostridia* bacterium]	MBQ1272506.1	68.23%
DCF-bgl-20	Glycoside hydrolase family 1 protein [*Clostridia* bacterium]	MBQ4323209.1	98.37%
DCF-bgl-21	Beta-glucosidase [*Clostridia* bacterium]	MBO7345591.1	68.15%
DCF-bgl-22	Beta-glucosidase [*Clostridia* bacterium]	MBQ8740605.1	72.28%
DCF-bgl-23	Beta-glucosidase [*Oscillospiraceae* bacterium]	MBR3978613.1	72.22%
DCF-bgl-24	Beta-glucosidase [*Oscillospiraceae* bacterium]	MBR3978613.1	71.78%
DCF-bgl-25	Beta-glucosidase [*Clostridia* bacterium]	MBQ9846934.1	99.78%
DCF-bgl-26	Beta-glucosidase [*Clostridia* bacterium]	MBQ1284466.1	99.78%
DCF-bgl-27	Beta-glucosidase [*Clostridia* bacterium]	MBQ9846934.1	97.57%
DCF-bgl-28	Beta-glucosidase [*Enterococcus casseliflavus*]	VTT36984.1	75.32%
DCF-bgl-29	Glycoside hydrolase family 1 protein [*Turicibacter* sp.]	MBQ1787025.1	99.57%
DCF-bgl-30	GH1 family beta-glucosidase [*Sarcina* sp. DSM 11001]	WP_092994021.1	99.35%
DCF-bgl-31	Family 1 glycosyl hydrolase [*Lachnospiraceae* bacterium]	MBR3186943.1	86.30%
DCF-bgl-32	Beta-glucosidase [*Oscillospiraceae* bacterium]	MBE6980119.1	77.17%
DCF-bgl-33	Beta-glucosidase [*Roseburia* sp.]	HAT88334.1	74.84%

## Data Availability

All data generated or analyzed during this study are included in this published article and its Supplementary Information files.

## Supplementary Materials

The following supporting information can be downloaded at: https://www.mdpi.com/article/10.3390/molecules29225280/s1, Figure S1: Screening of 13 putative β-glucosidases by LB-esculin-agar; Figure S2: Sequence comparison of DCF-bgl-26 and DCF-bgl-27 with another four GH1 family β-glucosidases; Table S1: The primers of 33 predicted β-glucosidase genes used for gene expression.
