# Supplementary material for: Efficient Biotransformation of Icariin to Baohuoside I Using Two Novel GH1 β-Glucosidases
Source: Molecules. 2024 Nov 8;29(22):5280. doi: 10.3390/molecules29225280 (PMC11596834; doi:10.3390/molecules29225280)
Supplement: Supplementary file 1 [file molecules-29-05280-s001.zip › molecules-3287747-supplementary.pdf]

Supplementary Information

The efficient biotransformation of icariin to baohuoside I using two novel GH1 β-glucosidases

Xiaoling Zhang[1,2,3,†], Yitong Wang[1†], Tiantian Zhang[1], Ziqiao Yuan[1*], Yongjun Wei[1,2,3*]

[1] School of Pharmaceutical Sciences, Zhengzhou University, Zhengzhou 450001, China

[2] State Key Laboratory of Cotton Bio-breeding and Integrated Utilization, Zhengzhou University, Zhengzhou 450001, China

[3] Food Laboratory of Zhongyuan, Zhengzhou University, Zhengzhou 450001, China

* Corresponding authors:

Ziqiao Yuan

figaroyzq@163.com

Yongjun Wei

yongjunwei@zzu.edu.cn

[†] These authors contributed equally to this work.

Supplementary tables and figures

Table S1 The primers of 33 predicted β-glucosidase genes used for gene expression.

Figure S1 Screening of 13 putative β-glucosidases by LB-esculin-agar.

Figure S2. Sequence comparison of DCF-bgl-26 and DCF-bgl-27 with another four GH1 family β-glucosidases. Br2: a β-glucosidase derived from bovine rumen metagenome (PDB ID: 8J3M); CcBglA: a β-glucosidase of *Clostridium cellulovorans* (PDB ID: 3AHX); TmGH1: a β-glucosidase of *Thermotoga maritima* (PDB ID:1OD0); IagBgl1: an β-glucosidase of *Ignisphaera aggregans*. The black asterisk indicated the two special strictly conserved motifs of GH1 β-glucosidases, the green triangles indicated the conserved amino acid residues in the substrate binding site of GH1 β-glucosidase, and the red stars highlighted the two predicted catalytic residues.

Table S1. The primers of 33 predicted β-glucosidase genes used for gene expression.

Primer names	Primer sequences
DCF-bgl-1-F	GCCTGGTGCCGCGCGGCAGCTTGCCATACGCATTGTATCTAAAAGG
DCF-bgl-1-R	GGTGCTCGAGTGCGGCCGCCAAATTTTCCCCGTTTGATTCAATCAC
DCF-bgl-2-F	GCCTGGTGCCGCGCGGCAGCATGAAATTTACTCTTAAAGACGGCCTT
DCF-bgl-2-R	GGTGCTCGAGTGCGGCCGCGTAGAATTTACCGCTGTTCTTCAC
DCF-bgl-3-F	GCCTGGTGCCGCGCGGCAGCATGACAGATATATTTCGCTGAAAGATTTT
DCF-bgl-3-R	GGTGCTCGAGTGCGGCCGCTTTTAAGCCGATTGACGGAATCG
DCF-bgl-4-F	GCCTGGTGCCGCGCGGCAGCATGTCAAAATTTCCAAAAGGATTTTTAATA
DCF-bgl-4-R	GGTGCTCGAGTGCGGCCGCTTTATTATATTCTCCCAGCAGATAAA
DCF-bgl-5-F	GCCTGGTGCCGCGCGGCAGCATGAAGTTTAAGCTTAAAGAGGGCATTC
DCF-bgl-5-R	GGTGCTCGAGTGCGGCCGCTAAGTACTGCGAATCCTTTACGAATTC
DCF-bgl-6-F	GCCTGGTGCCGCGCGGCAGCATGTTTACAACCCGTCTGATGAAAGAA
DCF-bgl-6-R	GGTGCTCGAGTGCGGCCGCGGAGCCTTTCTTGATCGGGAAA
DCF-bgl-7-F	GCCTGGTGCCGCGCGGCAGCATGAGCAAATTTTTAAAGGATTTCCTGTTG
DCF-bgl-7-R	GGTGCTCGAGTGCGGCCGCTTCTCCTTTGCCAAAGGAGCCA
DCF-bgl-8-F	GCCTGGTGCCGCGCGGCAGCATGCTTTCGGTCCTTTCCATGAAA
DCF-bgl-8-R	GGTGCTCGAGTGCGGCCGCGGAACCCTTCTTGATGGGCA
DCF-bgl-9-F	GCCTGGTGCCGCGCGGCAGCATGGGATTTTCAAAGGACTTTATGTGG
DCF-bgl-9-R	GGTGCTCGAGTGCGGCCGCGAGGTTCTCACCGTTGGTTTC
DCF-bgl-10-F	GCCTGGTGCCGCGCGGCAGCATGGCTTTTCCGAAAGATTTTCTTTGG
DCF-bgl-10-R	GGTGCTCGAGTGCGGCCGCTAAATTTTCCCCATTTGTTTTAATAACTTCCG
DCF-bgl-11-F	GCCTGGTGCCGCGCGGCAGCATGGCTTTTCCAAAGGACTTTTTATGG
DCF-bgl-11-R	GGTGCTCGAGTGCGGCCGCTTTGTGCGCTTTGATAAAATCCTTGTAG
DCF-bgl-12-F	GCCTGGTGCCGCGCGGCAGCATGAAATATGAAATTAGATTCGATTTTCCA
DCF-bgl-12-R	GGTGCTCGAGTGCGGCCGCGTCTACCCTGTTTTCTTTAATAACG
DCF-bgl-13-F	GCCTGGTGCCGCGCGGCAGCATGCCATTCCCGAAAAATTTCCTTTG
DCF-bgl-13-R	GGTGCTCGAGTGCGGCCGCCAGGCCTCTCGCCTTTTTGAT
DCF-bgl-14-F	GCCTGGTGCCGCGCGGCAGCATGCCTTTTCCTAAGAATTTCCTCTG
DCF-bgl-14-R	GGTGCTCGAGTGCGGCCGCTTTGGCGTTTTCTCGGACTTTTTCAA
DCF-bgl-15-F	GCCTGGTGCCGCGCGGCAGCGTGAAAATATCAATAATATTAAATTTAACTCC
DCF-bgl-15-R	GGTGCTCGAGTGCGGCCGCAAAACCATTGTTTTCTGAAACTGTTTC
DCF-bgl-16-F	GCCTGGTGCCGCGCGGCAGCATGGTTAAAAAGTTTAGTGAGAGGGG
DCF-bgl-16-R	GGTGCTCGAGTGCGGCCGCTAAGTTTTCGCCGTTCGTCTTGATTA

DCF-bgl-17-F	GCCTGGTGCCGCGCGGCAGCATGGCAATTATACAGTTTCCAAAAGACT
DCF-bgl-17-R	GGTGCTCGAGTGCGGCCGCCAATGTAAATTCACCGTTCTGAATCG
DCF-bgl-18-F	GCCTGGTGCCGCGCGGCAGCATGATTACATTTGATTTCCAAAGAATTTT
DCF-bgl-18-R	GGTGCTCGAGTGCGGCCGCGTCTACGCAACAATCGTTAATTA
DCF-bgl-19-F	GCCTGGTGCCGCGCGGCAGCATGAAATTTGAGTTTCCAAAGGACTTCT
DCF-bgl-19-R	GGTGCTCGAGTGCGGCCGCATTGACTCTTCCTTGCTCAATTACTTG
DCF-bgl-20-F	GCCTGGTGCCGCGCGGCAGCATGAAATTTCCGAAGGAATTTGTTTGGG
DCF-bgl-20-R	GGTGCTCGAGTGCGGCCGCTACTTCTCCGCTCCGGATGA
DCF-bgl-21-F	GCCTGGTGCCGCGCGGCAGCATGTCATTTCAAAAAAATTTGCATGGGG
DCF-bgl-21-R	GGTGCTCGAGTGCGGCCGCATGCGTTTTAATCCATTCCTTATACCAG
DCF-bgl-22-F	GCCTGGTGCCGCGCGGCAGCATGGCATTCAAAAAGGATTTCATTTGG
DCF-bgl-22-R	GGTGCTCGAGTGCGGCCGCTTTGCTGTGCTGTTCGATCCAAT
DCF-bgl-23-F	GCCTGGTGCCGCGCGGCAGCATGAGCTTCAAGAAAGGCTTTACCT
DCF-bgl-23-R	GGTGCTCGAGTGCGGCCGCATTGCTGTGATCTTCGATCCAAGA
DCF-bgl-24-F	GCCTGGTGCCGCGCGGCAGCATGAGCTTCAAAAAGGGTTTTACCTG
DCF-bgl-24-R	GGTGCTCGAGTGCGGCCGCGCAATTATGTGCTTCAATCCATGACT
DCF-bgl-25-F	GCCTGGTGCCGCGCGGCAGCATGTTTAGCAAGGACTTTGTCTGG
DCF-bgl-25-R	GGTGCTCGAGTGCGGCCGCTAGATTTTCACCGTTTGATGCAATAACA
DCF-bgl-26-F	GCCTGGTGCCGCGCGGCAGCATGTTCGGTAAAAATTTTGTTTGGGGAG
DCF-bgl-26-R	GGTGCTCGAGTGCGGCCGCAAGATTTTCGCCGTTAGTAGCGATTA
DCF-bgl-27-F	GCCTGGTGCCGCGCGGCAGCATGTTTTGTAAGGATTTTATCTGGGGTG
DCF-bgl-27-R	GGTGCTCGAGTGCGGCCGCAAGGGTTTCTCCGTTTGATGCAA
DCF-bgl-28-F	GCCTGGTGCCGCGCGGCAGCATGAATTACAAATTTCCAGCTGACTTCT
DCF-bgl-28-R	GGTGCTCGAGTGCGGCCGCGAAGCCGTTATTATCAGCGACTTC
DCF-bgl-29-F	GCCTGGTGCCGCGCGGCAGCATGAGTTTAAAGCATCAATTTCCTACTG
DCF-bgl-29-R	GGTGCTCGAGTGCGGCCGCAAAGCCATTATGCTGTGTTAACTGTTTA
DCF-bgl-30-F	GCCTGGTGCCGCGCGGCAGCATGCAGTTTAGTGATAAATTCGTATGGG
DCF-bgl-30-R	GGTGCTCGAGTGCGGCCGCGATGTTTTTTCCATTCGTCCGGATC
DCF-bgl-31-F	GCCTGGTGCCGCGCGGCAGCATGCAGTTTTCTGATCATTTTGTCTGG
DCF-bgl-31-R	GGTGCTCGAGTGCGGCCGCAATGATTCCCCCCTTGGTACGTA
DCF-bgl-32-F	GCCTGGTGCCGCGCGGCAGCATGGGCTTTCCCAAAGATTTTCTGT
DCF-bgl-32-R	GGTGCTCGAGTGCGGCCGCTGCGAGGTCCTCACCGTTG
DCF-bgl-33-F	GCCTGGTGCCGCGCGGCAGCATGAACACAAAAAGAGAAGAAAGATTATTTCC
DCF-bgl-33-R	GGTGCTCGAGTGCGGCCGCTACTGTTGAAGCAATAATTTCCATCTTTC

The underlined sequences of each primer are the overlap sequences that used for homologous recombination with the pET-28a (+) vector.

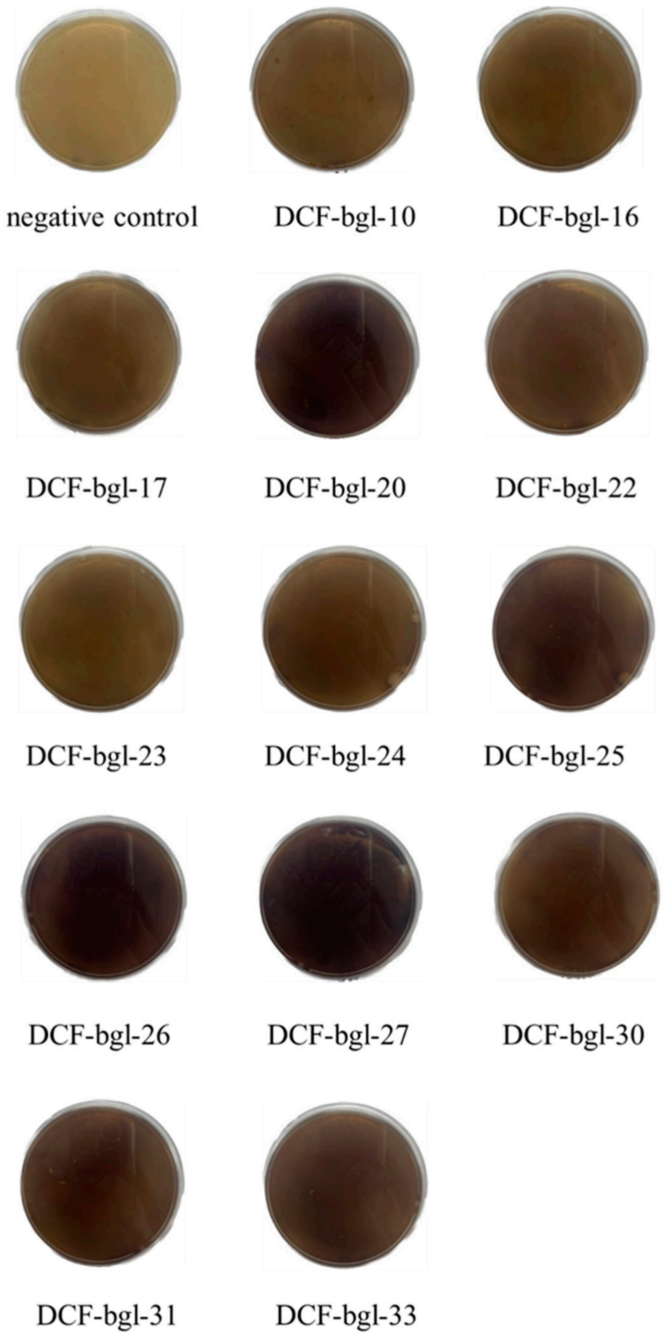

Figure S1. Screening of 13 putative β-glucosidases by LB-esculin-agar.

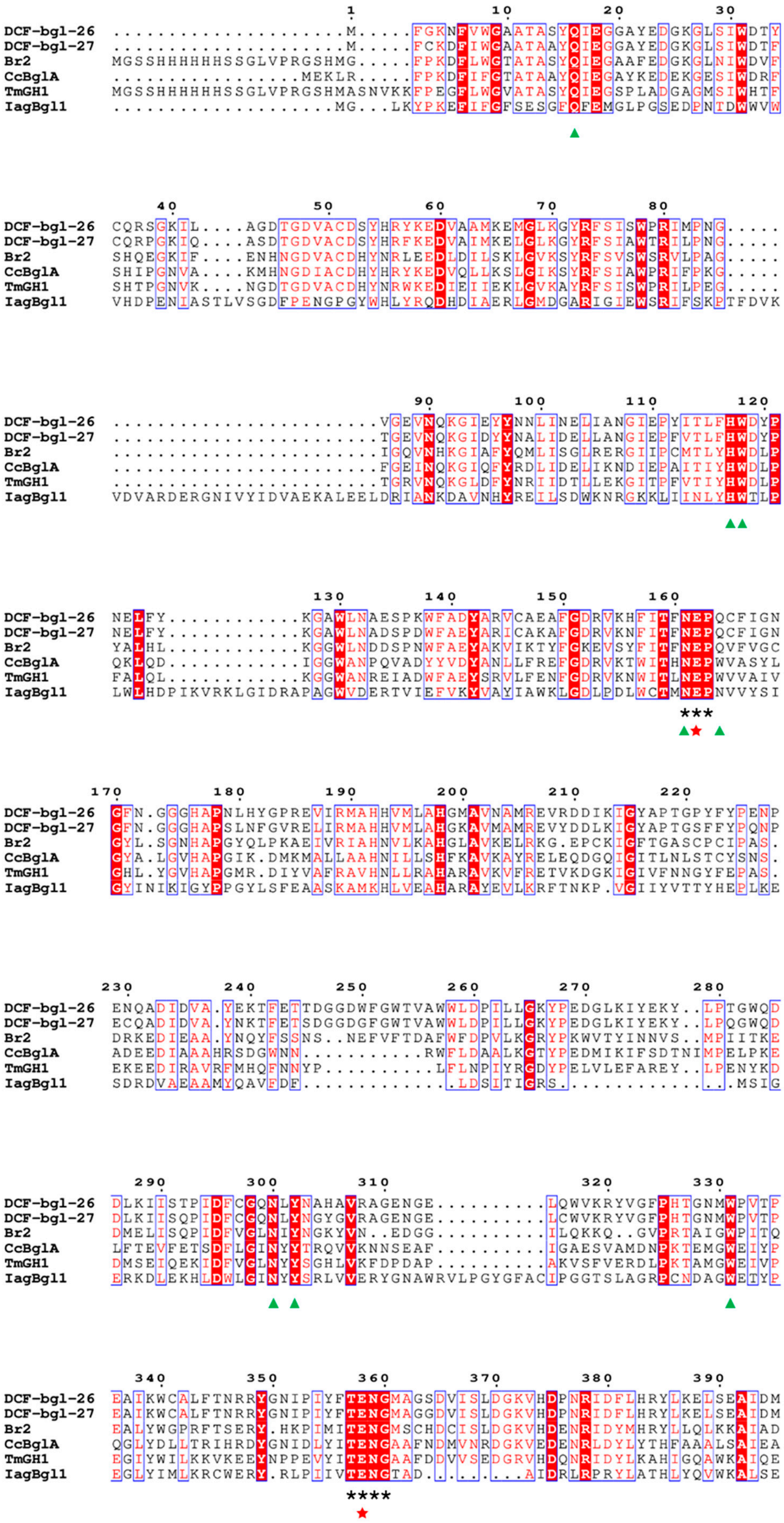

1 10 20 30
DCF-bgl-26 ....M.....FGKNFVWGAATASYQIEGGAYEDGKGLSIWDTY
DCF-bgl-27 ....M.....FCKDFIWGAATAAYQIEGGAYEDGKGLSIWDTF
Br2 MGSSHHHHHHSSGLVPRGSHMG.....FPKDFLWGTATASYQIEGAAFEDGKGLNIWDVF
CcBglA ....MEKLR.....FPKDFIFGTATAAYQIEGAYKEDEKGESIWDRF
TmGH1 MGSSHHHHHHSSGLVPRGSHMASNVKKFPEGFLWGVATASYQIEGSPLADGAGMSIWHTF
IagBgl1 ....MG...LKYPKEFIFGFSESGFQFEMGLPGSEDPNTDWWVW
40 50 60 70 80
DCF-bgl-26 CQRSGKIL....AGDTGDVACDSYHRYKEDVAAMKEMGLKGYRFSISWPRIMPNG.....
DCF-bgl-27 CQRPGKIQ....ASDTGDVACDSYHRFKEDVAIMKELGLKGYRFSIAWTRILPNG.....
Br2 SHQEGKIF....ENHNGDVACDHYNRLEEDLDILSKLGVKSYRFSVSWSRVLPAG.....
CcBglA SHIPGNVA....KMHNGDIACDHYHRYKEDVQLLKSLGIKSYRFSIAWPRIFPKG.....
TmGH1 SHTPGNVK....NGDTGDVACDHYNRWKEDIEIIEKLGVKAYRFSISWPRILPEG.....
IagBgl1 VHDPENIASTLVSGDFPENGPGYWHLYRQDHDIAERLGMDGARIGIEWSRIFSKPTFDVK
90 100 110 120
DCF-bgl-26 ......................VGEVNQKGIEYYNNLINELIANGIEPYITLFHWDYP
DCF-bgl-27 ......................TGEVNQKGIDYYNALIDELLANGIEPFVTLFHWDYP
Br2 ......................IGQVNHKGIAFYQMLISGLRERGIIPCMTLYHWDLP
CcBglA ......................FGEINQKGIQFYRDLIDELIKNDIEPAITIYHWDLP
TmGH1 ......................TGRVNQKGLDFYNRIIDTLLEKGITPFVTIYHWDLP
IagBgl1 VDVARDERGNIVYIDVAEKALEELDRIANKDAVNHYREILSDWKNRGKKLIINLYHWTLP
130 140 150 160
DCF-bgl-26 NELFY............KGAWLNAESPKWFADYARVCAEAFGDRVKHFITFNEPQCFIGN
DCF-bgl-27 NELFY............KGAWLNADSPDWFAEYARICAKAFGDRVKNFITFNEPQCFIGN
Br2 YALHL............KGGWLNDDSPNWFAEYAKVIKTYFGKEVSYFITFNEPQVFVGC
CcBglA QKLQD............IGGWANPQVADYYVDYANLLFREFGDRVKTWITHNEPWVASYL
TmGH1 FALQL............KGGWANREIADWFAEYSRVLFENFGDRVKNWITLNEPWVVAIV
IagBgl1 LWLHDPIKVRKLGIDRAPAGWVDERTVIEFVKYVAYIAWKLGDLPDLWCTMNEPNVVYSI
170 180 190 200 210 220
DCF-bgl-26 GFN.GGGHAPNLHYGPREVIRMAHHVMLAHGMAVNAMREVRDDIKIGYAPTGPYFYPENP
DCF-bgl-27 GFN.GGGHAPSLNFGVRELIRMAHHVMLAHGKAVMAMREVYDDLKIGYAPTGSFFYPQNP
Br2 GYL.SGNHAPGYQLPKAEIVRIAHNVLKAHGLAVKELRKG.EPCKIGFTGASCPCIPAS.
CcBglA GYA.LGVHAPGIK.DMKMALLAAHNILLSHFKAVKAYRELEQDGQIGITLNLSTCYSNS.
TmGH1 GHL.YGVHAPGMR.DIYVAFRAVHNLLRAHARAVKVFRETVKDGKIGIVFNNGYFEPAS.
IagBgl1 GYINIKIGYPPGYLSFEAASKAMKHLVEAHARAYEVLKRFTNKP.VGIIYVTTYHEPLKE
230 240 250 260 270 280
DCF-bgl-26 ENQADIDVA.YEKTFETTDGGDWFGWTVAWWLDPILLGKYPEDGLKIYEKY..LPTGWQD
DCF-bgl-27 ECQADIDVA.YNKTFETSDGGDGFGWTVAWWLDPILLGKYPEDGLKIYEKY..LPQGWQD
Br2 DRKEDIEAA.YNQYFSSNS..NEFVFTDAFWFDPVLKGRYPKWVTYINNVS..MPIITKE
CcBglA ADEEDIAAAHRSDGWNN...........RWFLDAALKGTYPEDMIKIFSDTNIMPELPKE
TmGH1 EKEEDIRAVRFMHQFNNYP..........LFLNPIYRGDYPELVLEFAREY..LPENYKD
IagBgl1 SDRDVAEAAMYQAVFDF..............LDSITIGRS...............MSIG
290 300 310 320 330
DCF-bgl-26 DLKIISTPIDFCGQNLYNAHAVRAGENGE..........LQWVKRYVGFPHTGNMWPVTP
DCF-bgl-27 DLKIISQPIDFCGQNLYNGYGVRAGENGE..........LCWVKRYVGFPHTGNMWPVTP
Br2 DMELISQPIDFVGLNIYNGKYVN..EDGG..........ILQKKQ..GVPRTAIGWPITQ
CcBglA LFTEVFETSDFLGINYYTRQVVKNNSEAF..........IGAESVAMDNPKTEMGWEIYP
TmGH1 DMSEIQEKIDFVGLNYYSGHLVKFDPDAP..........AKVSFVERDLPKTAMGWEIVP
IagBgl1 ERKDLEKHLDWLGINYYSRLVVERYGNAWRVLPGYGFACIPGGTSLAGRPCNDAGWETYP
340 350 360 370 380 390
DCF-bgl-26 EAIKWCALFTNRRYGNIPIYFTENGMAGSDVISLDGKVHDPNRIDFLHRYLKELSEAIDM
DCF-bgl-27 EAIKWCALFTNRRYGNIPIYFTENGMAGGDVISLDGKVHDPNRIDFLHRYLKELSEAIDM
Br2 EALYWGPRFTSERY.HKPIMITENGMSCHDCISLDGKVHDENRIDYMHRYLLQLKKAIAD
CcBglA QGLYDLLTRIHRDYGNIDLYITENGAAFNDMVNRDGKVEDENRLDYLYTHFAAALSAIEA
TmGH1 EGIYWILKKVKEEYNPPEVYITENGAAFDDVVSEDGRVHDQNRIDYLKAHIGQAWKAIQE
IagBgl1 EGLYIMLKRCWERY.RLPIIVTENGTAD.........AIDRLRPRYLATHLYQVWKALSE

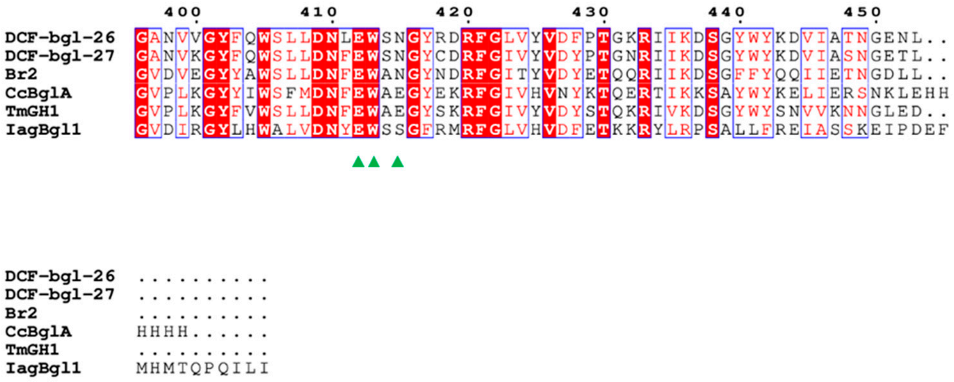

Figure S2. Sequence comparison of DCF-bgl-26 and DCF-bgl-27 with another four GH1 family β-glucosidases. Br2: a β-glucosidase derived from bovine rumen metagenome (PDB ID: 8J3M); CcBglA: a β-glucosidase of *Clostridium cellulovorans* (PDB ID: 3AHX); TmGH1: a β-glucosidase of *Thermotoga maritima* (PDB ID:1OD0); IagBgl1: an β-glucosidase of *Ignisphaera aggregans*. The black asterisk indicated the two special strictly conserved motifs of GH1 β-glucosidases, the green triangles indicated the conserved amino acid residues in the substrate binding site of GH1 β-glucosidase, and the red stars highlighted the two predicted catalytic residues.
